# An omni-channel, outcomes-focused approach to scale digital health interventions in resource-limited populations: a case study

**DOI:** 10.3389/fdgth.2023.1007687

**Published:** 2023-08-25

**Authors:** Aditi Hazra-Ganju, Schenelle Dayna Dlima, Sonia Rebecca Menezes, Aakash Ganju, Anjali Mer

**Affiliations:** Saathealth, Mumbai, India

**Keywords:** digital health, mobile health, mobile app, behaviour change, resource-limited settings, scaling

## Abstract

Populations in resource-limited communities have low health awareness, low financial literacy levels, and inadequate access to primary healthcare, leading to low adoption of preventive health behaviours, low healthcare-seeking behaviours, and poor health outcomes. Healthcare providers have limited reach and insights, limiting their ability to design relevant products for resource limited settings. Our primary preventive health intervention, called the *Saathealth* family health interventions, is a scaled digital offering that aims to improve knowledge levels on various health topics, nudge positive behaviour changes, and drive improved health outcomes. This case study presents our learnings and best practices in scaling these digital health interventions in resource-limited settings and maximising their impact. We scaled the *Saathealth* interventions to cumulatively reach >10 million users across India using a multi-pronged approach: (1) ensuring localization and cultural relevance of the health content delivered through user research; (2) disseminating content using omni-channel approaches, which involved using diverse content types and multiple digital platforms; (3) using iterative product features such as gamification and artificial intelligence-based (AI-based) predictive models; (4) using real-time analytics to adapt the user's digital experience by using interactive content to drive them towards products and services and (5) experiments with sustainability models to yield some early successes. The Saathealth family health mobile app had >25,000 downloads and the intervention reached >873,000 users in India every month through the mobile app, Facebook, and Instagram combined, from the time period of February 2022 to January 2023. We repeatedly observed videos and quizzes to be the most popular content types across all digital channels being used. Our AI-based predictive models helped improve user retention and content consumption, contributing to the sustainability of the mobile apps. In addition to reaching a high number of users across India, our scaling strategies contributed to deepened engagement and improved health-seeking behaviour. We hope these strategies help guide the sustainable and impactful scaling of mobile health interventions in other resource-limited settings.

## 1. Introduction

The growing community of urban underserved families in India is deprived of acceptable levels of health, nutrition, and preventive health information that is crucial to ensure positive health outcomes (1). Additionally, the awareness around financial literacy and its impact on health behaviours is low (2). Thus, there is a need to improve access to primary healthcare information and services among India's underserved population. The role of digital tools to expand the reach and access to timely health information is being increasingly recognized to help bridge the demand–supply gap that exists among health consumers and primary healthcare workers (3).

India is estimated to host close to one billion smartphone internet users by 2026, and higher internet adoption in rural areas is expected to propel the sale of internet-enabled phones (4). Monthly mobile data consumption per user is growing at 152% annually, more than twice the rates in the United States and China (5). The almost ubiquitous presence of mobile phones makes them powerful channels for imparting health information and expanding its reach in a cost-effective, scalable manner, even in resource-limited communities. The COVID-19 pandemic also propelled an increased use of digital health tools, such as for communication and information, monitoring and surveillance, supporting provision of healthcare, and roll-out of vaccinations (6, 7). Thus, digital tools are growing in accessibility and usability for Indian consumers, further driven by the wide-spread use of digital health tools during the pandemic.

The double burden of low health awareness and inadequate access to primary healthcare results in low prevalence of health-promoting behaviours and poor health outcomes. *Saathealth's* primary preventive health intervention is a scaled digital offering providing families with the behaviour change nudges they need at the right time and with a single touchpoint. This intervention was initially launched as a children's health app in 2018 to meet the childhood development information needs of urban, low-income parents in India. Through the *Saathealth* mobile app, our objective was to connect digital India with a mobile health (mHealth) intervention that could address the most common gaps in health awareness and practices in resource-limited settings.

The *Saathealth* children's health intervention achieved >100,000 downloads and reached >54,000 families through the mobile app, Facebook, and YouTube across India from August 2018 to January 2021. In 2022, the app expanded its scope, content library, and product offerings to serve the entire family's health needs. This is because health needs evolved since the COVID-19 pandemic and families need tools that allow them to take charge of their health decisions. Moreover, information needs in other health and disease areas, such as non-communicable diseases (NCDs) and mental health, took a backseat during the pandemic, resulting in the widening of health information gaps (8). NCDs pose a significant public health burden in low- and middle-income countries (LMICs); approximately 85% of premature deaths due to NCDs occur in LMICs (9). In India, NCDs are the leading causes of premature death and accounted for 61.8% of premature mortality in 2016, with cardiovascular disease, chronic obstructive pulmonary disease, type 2 diabetes, and cancer contributing to the highest number of deaths (10). Hence, there are significant opportunities to address the behavioural risk factors for these common NCDs, such as unhealthy dietary practices, lack of exercise, tobacco use, and alcohol consumption, through credible health content delivered in engaging ways.

The cost of healthcare, which is usually an out-of-pocket expense in LMICs, is rapidly increasing and the public health policy changes and improvements are not able to address the burden on the system. Approximately 63% of the Indian population is not covered by any health insurance scheme. Health emergencies, including outpatient consultations, force Indians to pay out-of-pocket for health services, raising their risk of being pushed into poverty (11). A recent Niti Aayog policy document highlighted that lack of awareness and understanding of complex products such as health insurance, especially amongst the missing middle, hinder their adoption by clients (12). Consumer education and awareness campaigns as well as digital sales channels can bring down costs, such as commissions, incurred by insurance providers, hence making health insurance more affordable for end-clients.

In this case study, we present our learnings from our extensive mHealth deployments, focusing on family health interventions and describe our best practices to reach health consumers directly and in real time. We detail the multi-pronged approach used by the *Saathealth* intervention to deliver health content to millions of users in India and discuss how health content consumption via digital channels helps drive positive behaviour change. We hope this case study helps guide mHealth intervention development in resource-limited settings and demonstrate that scalability with improved outcomes can be achieved in such settings as well.

## 2. Materials and methods

**Figure 1:**
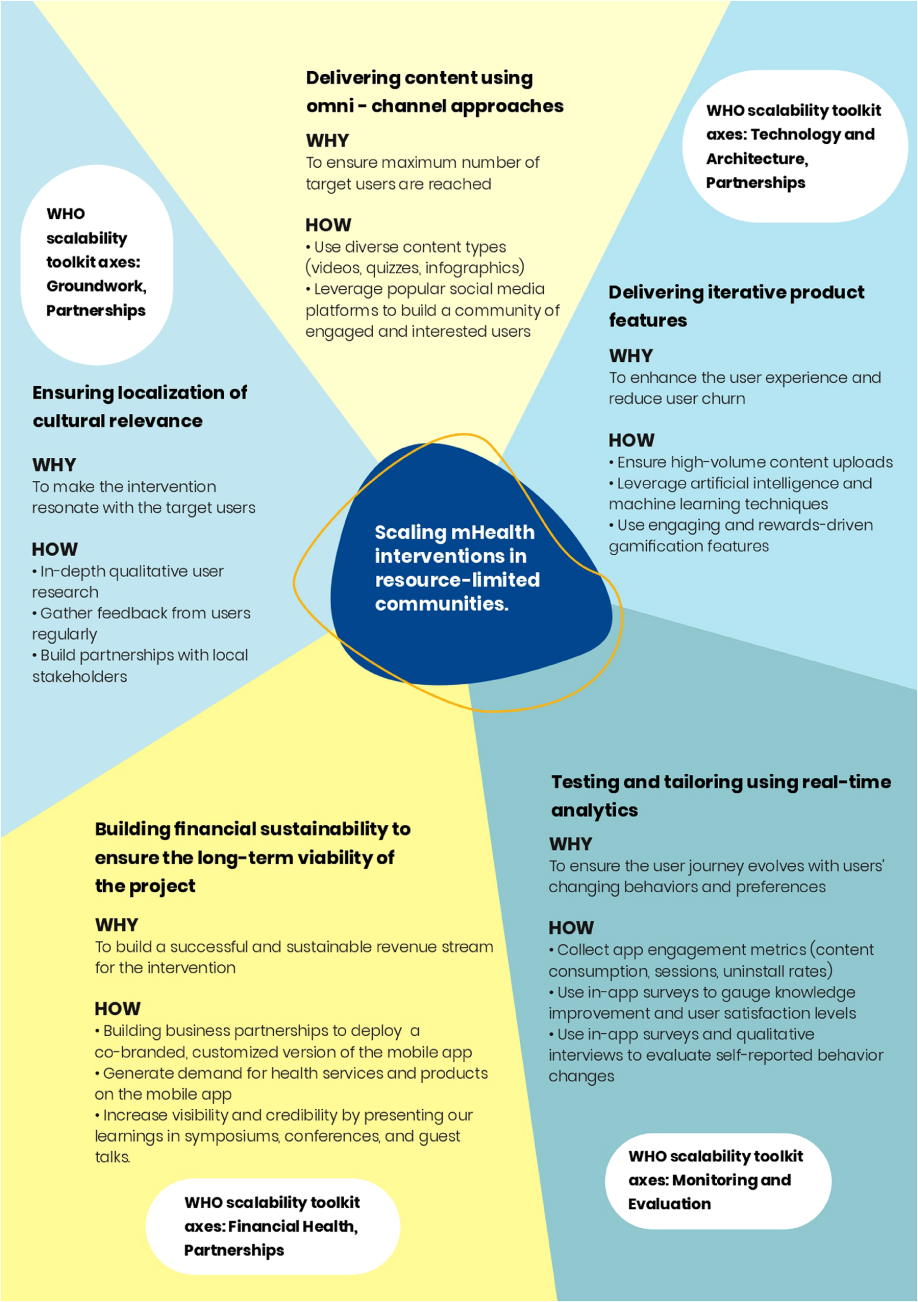
A visual representation of the most effective strategies used to scale the *Saathealth* children's and family health intervention in low-income populations in India. These strategies can help guide the scaling of mHealth interventions among target users in resource-limited communities.

We used a multi-pronged approach to reach millions of users across India with the *Saathealth* family health digital intervention. In this paper, we self-assess our multi-pronged approach against the World Health Organization's (WHO's) mHealth Assessment and Planning for Scale (MAPS) toolkit (13). The toolkit includes six “axes of scale” to self-evaluate the scalability and sustainability of mHealth interventions: groundwork, partnerships, financial health, technology and architecture, operations, and monitoring and evaluation. We highlight the execution of our most effective strategies against domains in the following axes: groundwork, partnerships, technology and architecture, and monitoring and evaluation. We focused primarily on four of the six axes as they were most relevant to the multi-pronged approach used for the *Saathealth* interventions. Figure 1 presents a visual representation of our scaling strategies and how they align with the axes in the WHO mHealth MAPS toolkit.

### 2.1. Ensuring localization, contextual and cultural relevance

The *Saathealth* interventions offer Hindi-language content to address the dearth of credible health information in the vernacular. Our digital intervention was first launched as the *Saathealth* children's health mobile app in 2018, which delivered content and gamified quizzes on children's nutrition and early learning. During the COVID-19 pandemic, we realised that there was an opportunity to bridge the gap between health-seekers and health service providers by using a digital platform that was tailored to the needs of vernacular populations in India, to both inform and empower them with access to better health. These health service providers include clinicians and health insurance organisations. The *Saathealth* intervention was then modified and relaunched in 2022 with additional content and features to cater to the health needs of the entire family.

Our content is based on the Fogg Behavior Model, which posits that three elements must converge for a behaviour to occur: motivation, ability, and a prompt. The target user should be sufficiently motivated, able to perform the behaviour, and triggered to perform the behaviour (14). For example, earning points in the app was a motivator, users had the ability to redeem their points, and push notifications served as prompts. Our formative research helped us create features that motivated the target users to change their health behaviours (financial incentives for healthy purchases) and understand their context-specific ability to perform the behaviours.

**Figure 2:**
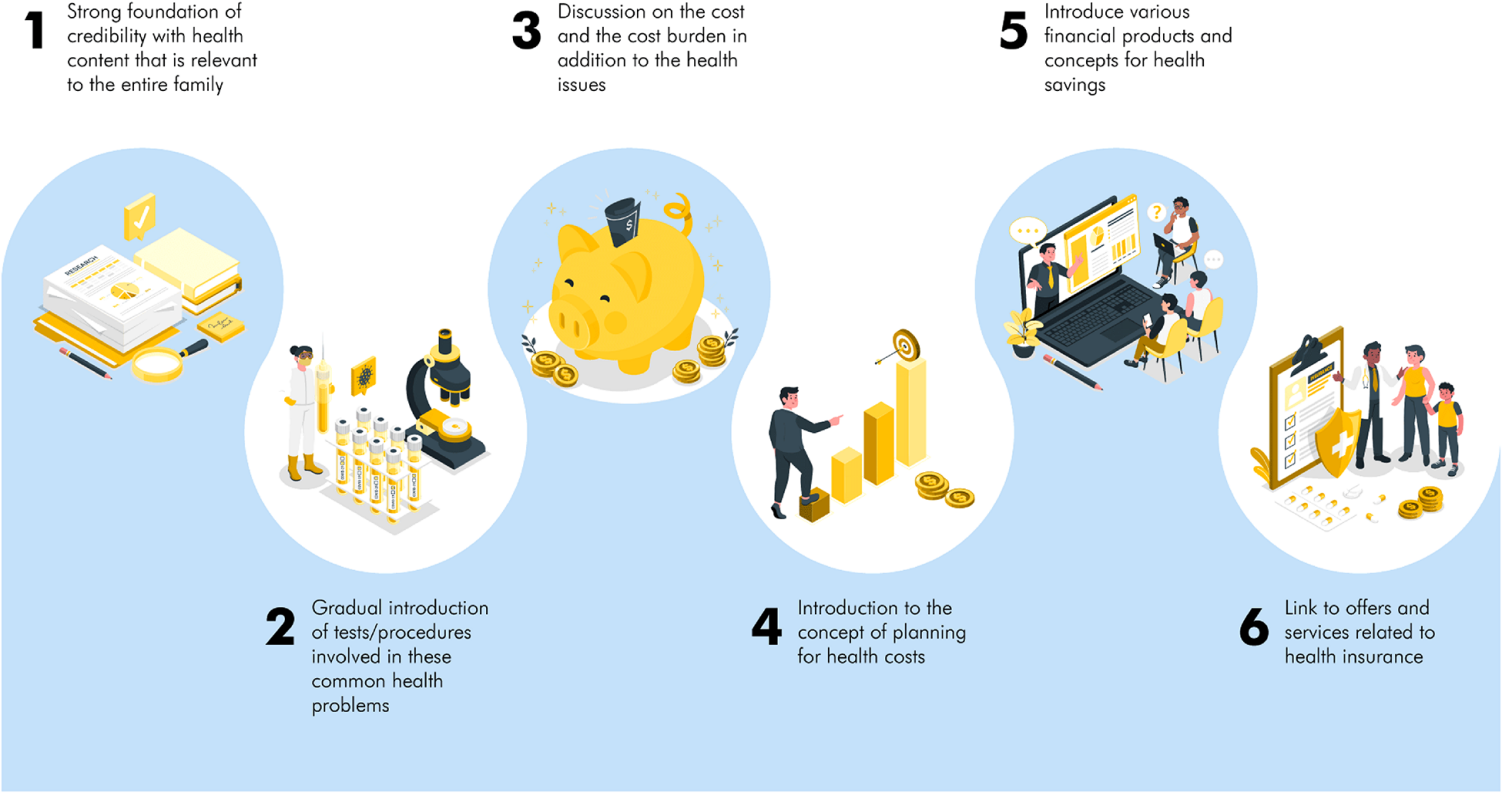
Cohesive content story that weaves the entire chain of information.

Our approach to use infotaining health content to engage communities to promote health savings awareness and generate demand for preventive health behaviours in different therapeutic areas, health services, financial products to serve health needs continued to be our focus in the last few months. The approach we followed towards a cohesive content story that weaves the entire chain of information is described in Figure 2.

As we expanded to an intervention to address family health, semi-structured interviews with target users helped us identify knowledge gaps and barriers in health-seeking behaviours. These interviews were conducted by members of the content team, and focused on knowledge-seeking behaviours, awareness, and current practices in family health. We conducted a financial literacy and health savings survey to better understand the health needs and expenses of families in low and middle income communities. The content map for the family health intervention focused on key health themes, and a detailed, evidence-based set of health messages was created on each theme, which was then used as a base to build the content library.

The localization of acquisition and incentivization in the children's health app is being explored for national scale-up in the family health intervention through multiple models. Furthermore, a crucial objective is to demonstrate financial sustainability models to monetize the intervention. This approach of ensuring that the mHealth intervention is culturally relevant and localised aligns with the domains of contextual environment and scientific bases in the groundwork axis of the WHO MAPS toolkit.

### 2.2. Disseminating content using omni-channel approaches

**Figure 3:**
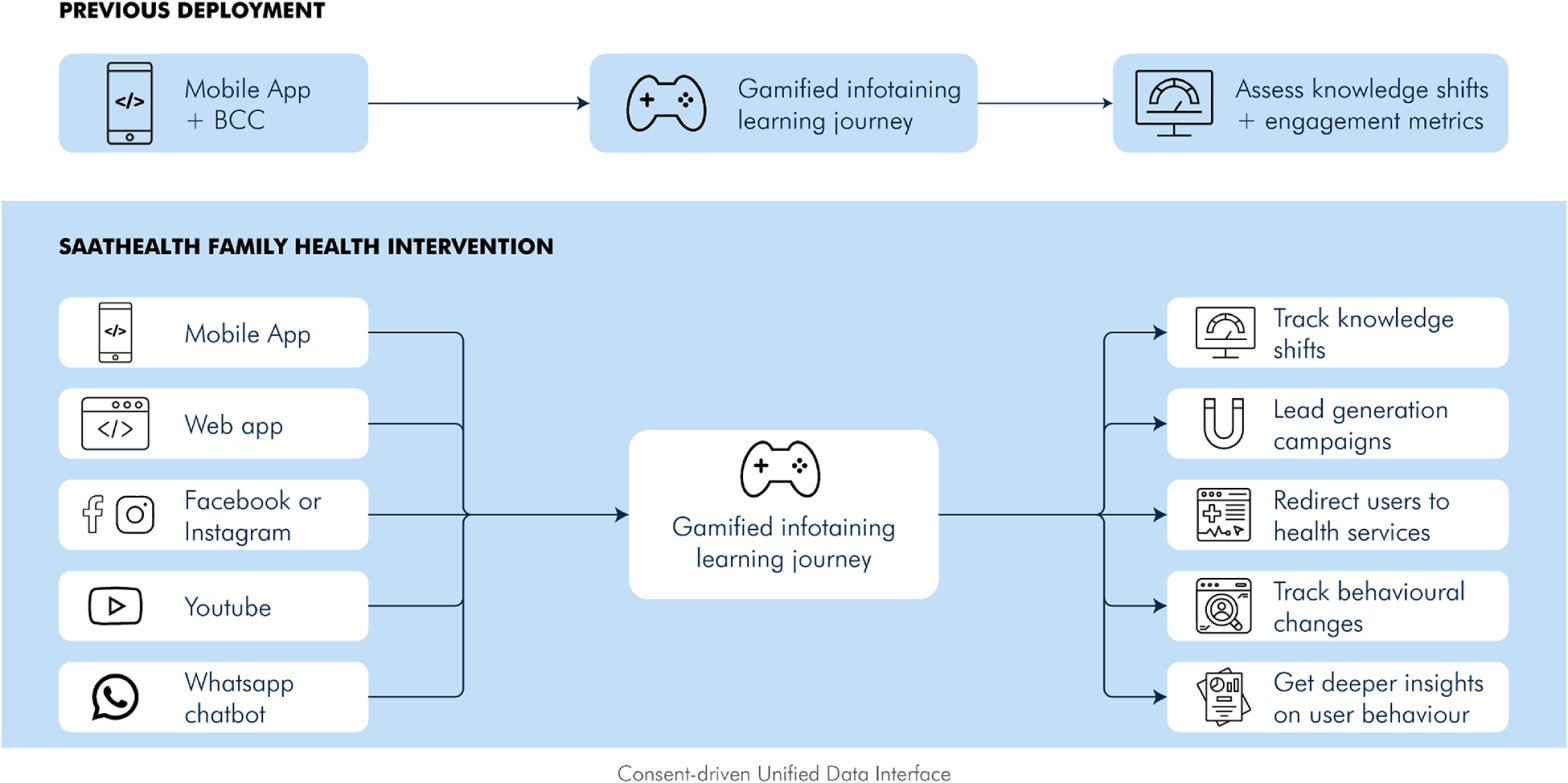
Dissemination of content using omni-channel approaches.

**Figure 4:**
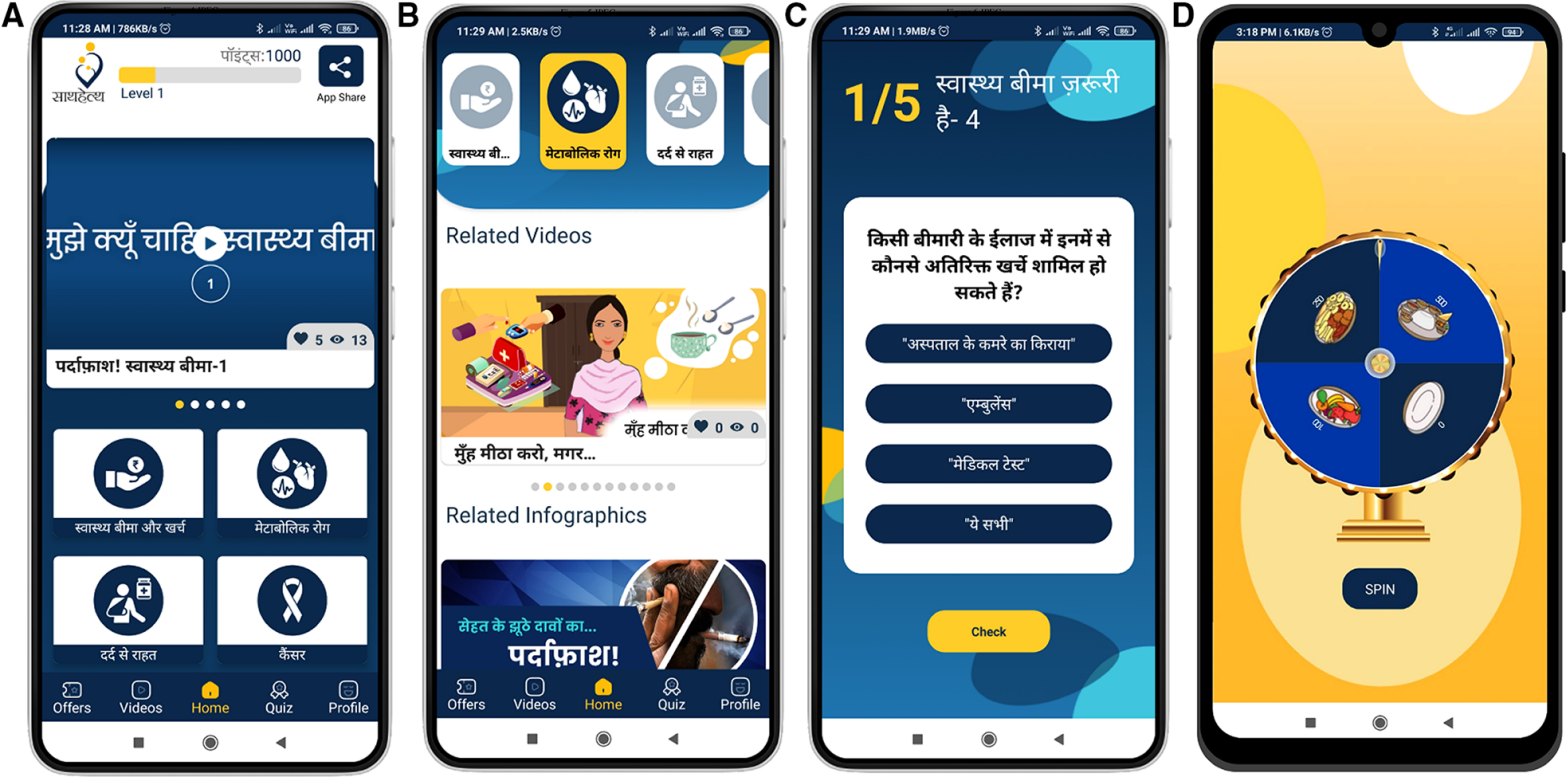
The presented figures exhibit the user interface and diverse content types of the Saathealth family health app. (**A**) Displays the distinct categories of content available within the application, with the upper banner depicting the user's earned points and a share app button. (**B**) Illustrates the manner in which videos are presented within the app, while (**C**) depicts a quiz question with options that the user can select from. (**D**) Demonstrates the gamification feature, “spin-the-wheel,” which permits users to earn points.

**Figure 5:**
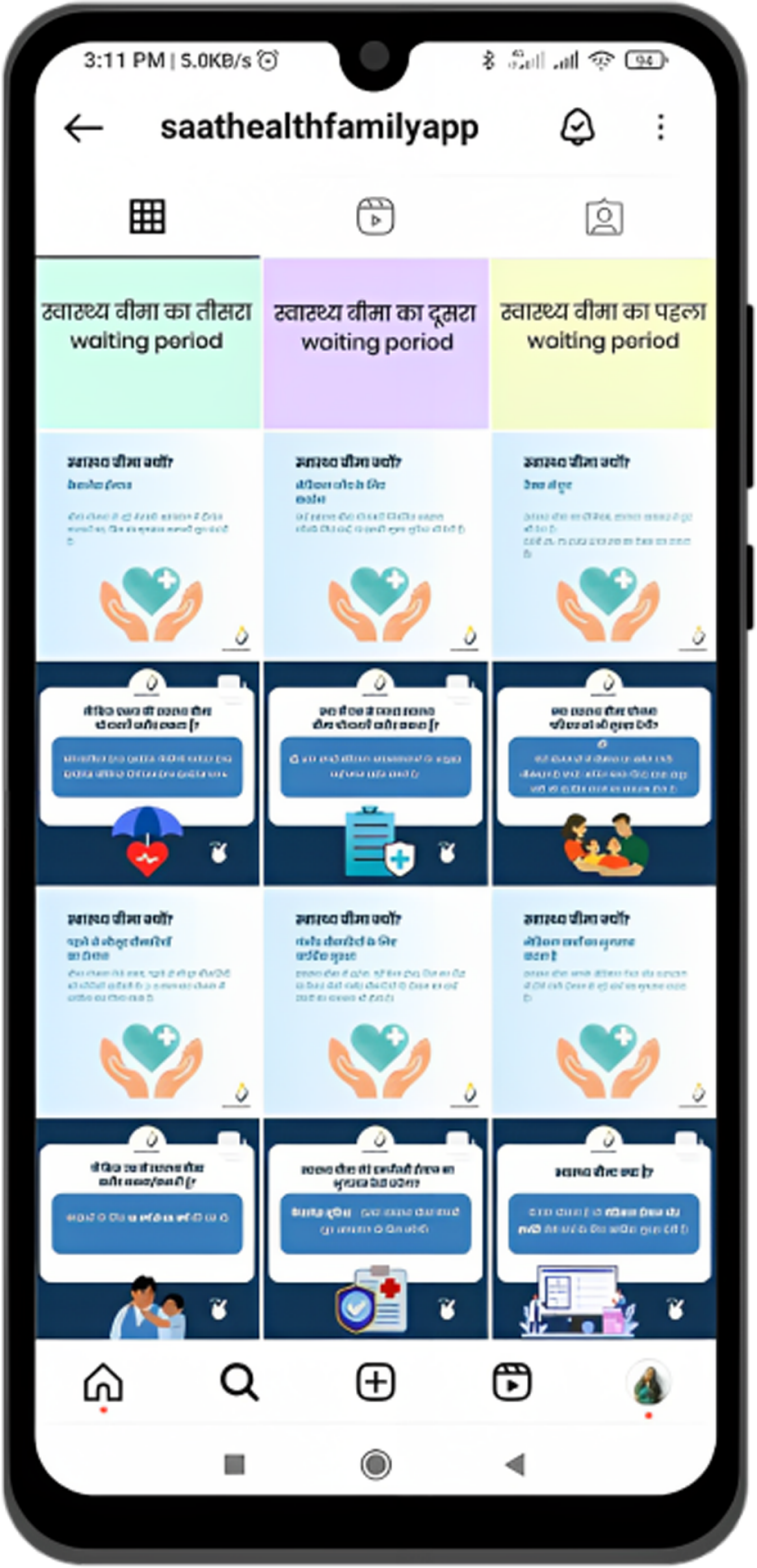
The screenshot illustrates the user interface and content types of the Saathealth family health intervention as displayed on the Instagram platform and depicts how posts are surfaced.

Users have different preferences when it comes to content consumption; hence, the *Saathealth* interventions deliver content in multiple formats to ensure maximum number of users are reached. Figure 3 depicts dissemination of content using omni-channel approaches. Our content types include animated video series with relatable characters, audio led video infographics, short gifs, reels, notifications, conversations with experts, and rewards-driven quizzes. In the family health intervention, we diversified our content types further and added content on several new health topics, including cardiovascular disease, type 2 diabetes, thyroid disorders, mental health, cancer, seasonal infectious diseases such as tuberculosis and typhoid, vector borne diseases, gastrointestinal disorders, diagnostic tests, essentials of hospitalisation, pain management and financial literacy. Figure 4 exhibits a series of screenshots that illustrate the various types of content accessible in the family health app. On the other hand, Figure 5 demonstrates an array of content types presented on the family health Instagram page. To enhance the content's credibility and to address specific health concerns communicated by users, we partnered with leading medical experts and created short videos where they address health concerns in an easily understandable manner. Based on the content engagement metrics that we regularly monitor, we gradually converted all static posts such as infographics to audio–visual content pieces using motion graphics and gifs. We gave a fun twist to our mythbuster content by creating a series called *Pardafaash* (which means “to reveal the truth”), which presents the myth busters in an audiovisual format with caricaturish voices.

To further widen our reach, we developed parallel user acquisition and engagement journeys using popular social media platforms such as Facebook, Instagram, YouTube. With the family health intervention, we started uploading reels on Instagram on different health topics we cover in the app. These reels are distinct from the animated and doctor-delivered videos found in the app; the reels aim to deliver succinct health messages, provide a sneak peek into the health content offered in the app, and nudge users to download the app. In addition to the in-app and push notifications, we expanded messaging outreach to the users using email newsletters and Facebook messenger, through which we were able to gather user feedback to improve and further contextualise our content. Our team is currently working on a WhatsApp chatbot journey, which when launched, will give us significantly deeper insights into our users' needs and allow us to iterate our product and service offerings accordingly.

### 2.3. Delivering iterative product features

To ensure user engagement with the app and improve content consumption, we used high-volume weekly content uploads and nudges. Our new weekly content uploads included two videos, 10 quiz questions, 10–12 push notifications, and 4–5 infographics. We often accompanied these with surprise bonus points at unexpected times with changes to the points logic on the app backend.

We also used AI and machine learning to deliver adaptive and personalised user journeys at scale. In the children's health intervention, we utilised app analytics from approximately 45,000 users to build machine learning-based models that could predict user churn, a metric used to quantify the number of users who uninstall an app in a specific time period. This work has been previously published (15). We also built machine learning-driven recommender systems to surface health content that the user is most likely to engage with (16).

We gamified the in-app learning journey using quizzes, spin-the-wheel games, and engaging notifications, and users could track their points journey vis-a-vis their peers via leaderboards and progress to the next level in the leaderboards. These motivational elements are now extended to our on-the-ground partner organisations, where in exchange for encouraging users to download the app, these organisations benefit from the services and products they provide to families. These could range from low-cost telehealth services to e-pharmacy products to financial products to manage healthcare costs.

The “share app” feature in the children's health mobile app was important in serving a dual purpose: increasing the virality of the app and reducing the user acquisition cost. This feature allowed users to easily share the app with their friends and family. This feature also falls within the “Partnerships” axis of the WHO scalability toolkit, as the share app feature enabled users themselves to become “champions” who were able to advocate for the app to their friends and family.

We understand that the pandemic created more discerning health consumers, and one of the ways in which the *Saathealth* family health intervention used innovation to expand access to social channels was by pulling in the feed from our Instagram account into the app home page. This gave our existing app users a glimpse of a parallel source of content and helped us gain more followers for our Instagram channel.

### 2.4. Real-time analytics to adapt the digital experience to drive behaviour change

**Figure 6:**
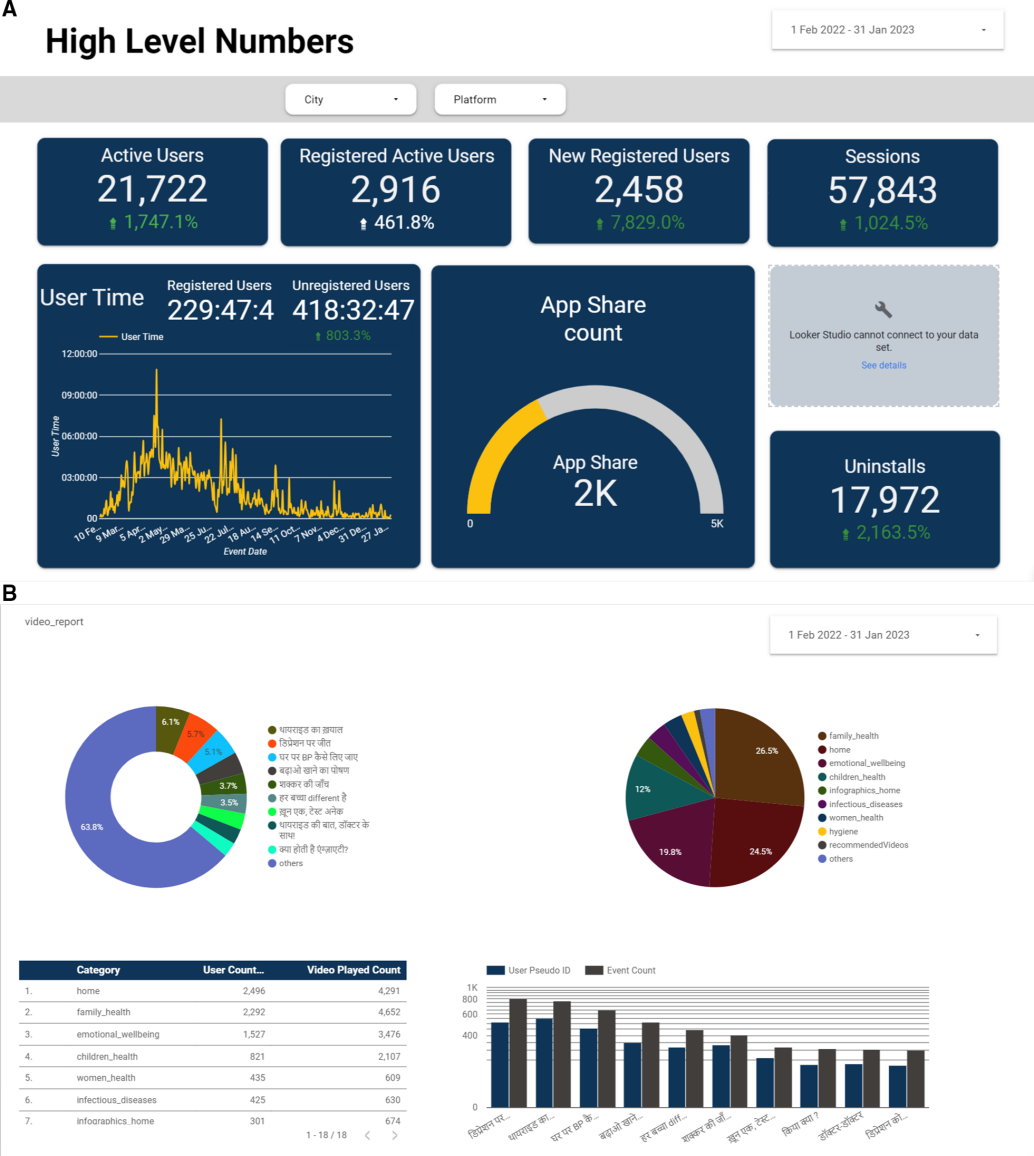
(**A,B**) Screenshots of the *Saathealth* family health app dashboard on Google data studio. Our dashboard presents data points, trends, and metrics in a visual and easily understandable format, enabling US to gain a quick overview of real-time app engagement levels.

Using active and passive data collected from the app, we measure app engagement metrics, health behaviour shifts, and knowledge shifts to inform our content approach and product changes. Our live dashboard, as seen in Figures 6A,B, captures granular data related to new users onboarded, uninstall rates, content consumption, engagement with gamification elements, correct quiz responses, and in-app surveys. These real-time insights allow us to test and iterate new content types and themes, product changes, and gamification elements on a monthly basis, helping us drive users towards products and services. We also collect analytics from the social media platforms to assess reach numbers and content popularity. Table 1 lists all the data points that we capture from our live dashboard and social media platforms.

**Table 1:** A list of the data metrics collected by the *Saathealth* family health app dashboard and from the social media platforms.

Platform	Metric (unit)	Definition
*Saathealth* family health mobile app	Installs (*n*)	Number of new app installs from Google Play Store in a specific time period.
	Total cumulative users (*n*)	The total number of users (both registered and unregistered) on the app at a given point in time.
	New users (*n*)	Number of users who have installed and opened the app for the first time during a specific time period.
	Active users (*n*)	Number of users who opened the app during a specific time period.
	Total number of sessions (*n*)	Number of times all users spent >10 s on the app during a specific time period.
	Registrations initiated (*n*)	Number of times users started the registration process during a specific time period.
	Registrations (*n*)	Number of complete registrations during a specific time period.
	Sessions per active user	Total number of sessions/number of active users during a specific time period.
	Average session duration (s)	Sum of the durations of all sessions in a specific time period/total number of sessions in a specific time period
	Total in-app time by all users (s)	Total time spent on opening the app by all users in a specific time period.
	Videos watched (*n*)	Number of both complete and incomplete video watches in a specific time period.
	Quiz responses (*n*)	Number of quiz questions attempted by users in a specific time period.
	App shares (*n*)	Number of times the share app feature was used by users in a specific time period.
	Average engagement time (s)	Average time a user engaged with some content on the app.
	Daily active users (*n*)	Number of active users on a specific day.
	Monthly active users (*n*)	Number of active users in a given month.
	Uninstalls (*n*)	Number of times the app was uninstalled during a specific time period.
	Cost of acquisition per new user (INR)	Cost to acquire a new user via Google ad and social media campaigns.
Social media platforms	New content	Total number of content pieces uploaded on Instagram, Facebook, and YouTube in a specific time period.
	Total number of cumulative followers	Total number of followers or subscribers on Instagram, Facebook, and YouTube at a given point in time.
	Number of new followers	Number of new followers or subscribers on Instagram, Facebook, and YouTube in a specific time period.
	Total community interactions	This includes engagement with all Facebook posts and stories (likes, shares, views, comments, story responses on polls, and views) and with all Instagram posts, stories, reels, and IGTV videos (likes, comments, shares, saves, and video interactions).

These real-time analytics enabled us to create an autonomous user experience in which we optimised their content consumption and provided effective nudges to drive positive behaviour change. An example of how we did this was when we discovered that users spent maximum time on the app answering quiz questions when we monitored their content consumption patterns. We immediately doubled the number of quiz questions associated with each video so users could do more of what they enjoyed on the app. Similarly, when we observed a low uptake with content types that were not engaging for our users (using puppets instead of animated characters), we discontinued the use of that content format. We are employing the same iterative data analysis approaches for the family health app.

This “test-and-tailor” strategy falls within the “Monitoring and Evaluation” axis of the WHO scalability toolkit. Within the “process monitoring” domain, we conducted routine and on-going reviews of the app analytics and tracked implementation milestones to continue scaling the intervention. Within the “evaluation research” domain, we track the benefits of the *Saathealth* interventions on individual-level knowledge improvements and health behaviour changes using self-reported data in the app.

### 2.5. Building financial sustainability to ensure the long-term viability of the project

Building financial sustainability to ensure the long-term viability of the project is a key focus of the Saathealth family health intervention. By identifying methods to monetize our offerings and building meaningful partnerships with other organisations, we have been able to experiment with and build a successful revenue stream in the following ways:

• Building and deploying a co-branded, customised version of the app for effective and long-term engagement within specific communities
• User acquisition and demand generation for health insurance, health service and products.

In addition, we continue to work on gaining exposure and visibility by presenting our learnings from several successful pilots in symposiums, conferences, guest talks, etc.

Our efforts to ensure financial sustainability fall under the “financial health” and “partnerships” axes of the WHO scalability toolkit. As we launched our family health intervention, we refined our approach and explored long-term, sustainable financing opportunities by including health services in our digital interventions. We aim to diversify our funding sources to include both grant-based and long-term financial modes to ensure the financial health of our intervention.

## 3. Results

The *Saathealth* family health app has been available in the Google Play Store since February 2022. As of January 2023, the family health app has achieved >25,000 downloads and reached >873,000 users in India every month through the mobile app, targeted social media promotions on Facebook and Instagram, and posting content on Facebook and Instagram. The number of active users on the family health app increased by 17.5% (from 467 to 549 users) and the total number of cumulative users increased by 44.4% (from 51,995 to 75,086 users). The total reach using the social media channels increased by 375% (from 319,923 to 1,520,413 users). The subsequent sections detail how our scaling strategies for the *Saathealth* interventions helped increase and sustain content consumption, product engagement, health knowledge levels, health behaviour changes, and user satisfaction.

### 3.1. Content consumption on the *Saathealth* interventions

In the *Saathealth* children's health app, users received new content daily in multiple, consumable formats that included videos, infographics, notifications, and interactive quizzes. During the time period of July 2019 to September 2019, the Saathealth children's health app had 131,618 video watches (including incomplete watches), 12,501 infographic views, and 180,449 quiz questions attempted, with a notification open rate of 26.7%. In the family health app till January 2023, we have delivered 1,133 content pieces of new content related to family health topics in the form of animated videos, explainer videos by health experts, quizzes, dynamic infographics with voiceovers, notifications, creatives for lead generation campaigns, and social media reels. 8,517 users engaged with at least one content piece. Table 2 presents content consumption data on the *Saathealth* family health mobile app.

**Table 2:** Data on content consumption in the *Saathealth* family health mobile app.

App metric	*Saathealth* family health app	Time period
Video watches (including incomplete watches) (*n*)	11,422	February 2022 to January 2023
Infographic views (*n*)	7,373	February 2022 to January 2023
Quiz questions attempted (*n*)	10,952	February 2022 to January 2023
Notification open rates (%)	11.5%	February 2022 to January 2023

Based on the video watches in the family health intervention from February 2022 to January 2023, the three most popular health categories were “family health” (4,652 video watches), “emotional wellbeing” (3,476 video watches), and “children's health” (2,107 video watches).

The family health intervention covers a diverse range of topics and till date, we have uploaded family health content related to back pain, thyroid disease, chronic obstructive pulmonary disorder, mental health, diagnostic tests, type 2 diabetes, cardiovascular disease, cancer awareness, and financial literacy. Tables 3, 4 provides descriptions of the most frequently consumed content pieces on the *Saathealth* family health interventions from February 2022 to January 2023. These insights on the most popular content pieces helped inform our future content strategy.

**Table 3:** Details on the most frequently consumed content pieces in the *Saathealth* family health mobile app from February 2022 to January 2023.

Content type	Content title in Hinglish	English title	Content category	Description	Number of views/attempts
Video	*Depression Par Jeet*	Victory Over Depression	Emotional well-being	A trained psychologist gives advice on how to deal with stress and depression.	806
	*Thyroid ka khayal*	Taking care of Thyroid	Family health	A short step-by-step guide to take care of thyroid.	771
	*Ghar Par* BP *Kaise Liya Jaaye*	How To Measure Your BP At Home	Family health	A short step-by-step guide to home BP measurement.	653
Infographic	*Badhao khaane ka phoshan*	How to increase nutrition in food	Family health	A short step-by-step guide to check nutrition.	517
	*Har bhacha different hai*	Every child is different	Family health	A short video on how each child is different in health aspect.	448
	*Shakkar ka jhaanch*	Testing for blood sugar levels	Family health	An introduction on blood tests for tracking blood glucose levels.	402
Quiz	Depression *Par Jeet*	Victory Over Depression	Emotional well-being	Questions related to Depression to evaluate the knowledge retained	1,284
	*Kya Hota Hai* Depression?	What Is Depression?	Emotional well-being	Questions related to Depression to evaluate the knowledge retained.	622
	*Shakkar Ki Janch*	Testing for blood sugar levels	Family health	Questions related to checking for blood sugar levels to evaluate the knowledge retained.	740

BP, blood pressure.

**Table 4:** Details on the most frequently consumed content pieces in the *Saathealth* family health Instagram from February 2022 to January 2023.

Content type	Content title in Hinglish	English title	Content category	Description	Number of views/attempts
Reel	*Thyroid kya hai*	What is thyroid	Metabolic syndrome	A short video on importance of thyroid	2,929
Reel	*Anxiety hatao aise- 1*	Get rid of anxiety like this- 1	Emotional wellbeing	A short step-by-step guide to get rid of anxiety	2,896
Reel	*Vitamin Ki ABCD*	The ABCD of Vitamins	Metabolic syndrome	A short trick to remember the importance of each Vitamin.	1,612

In all our interventions, we repeatedly find that the most popular content types are audio–visual reels or videos. In the children's health intervention between July and September 2019, there were 185,810 video watches. In the family health intervention, the total number of video watches was 11,422 from February 2022 to January 2023.

### 3.2. Engagement with product features on the *Saathealth* interventions

We used AI and machine learning to predict user churn and to push relevant content to improve engagement in our health app. We achieved a 93% accuracy in predicting user churn and increased the average number of days-on-app from 38 to 85 through targeted messaging strategies (15). We also built health recommendation engines using machine learning-based content and collaborative filtering algorithms, which resulted in higher user engagement compared to those who did not receive personalised recommendations (16). The use of AI and machine learning to predict user behaviour and personalise recommendations can significantly improve user engagement and retention. This can have positive implications for any intervention looking to increase their user base and drive revenue through increased user activity and loyalty.

We introduced a new gamification feature, called spin-the-wheel, in the *Saathealth* family health mobile app in May 2022. Users can tap on the wheel and earn points instantly. As of January 2023, 4,150 times the spin-the-wheel game was initiated.

### 3.3. Improvements in knowledge levels and health-seeking behaviours

The *Saathealth* children's health intervention users demonstrated improved knowledge levels on topics related to child nutrition and parenting. After watching our video content on infectious diseases, the percentage of respondents that correctly answered how to prevent mosquito-borne diseases increased from 36.0% to 74.0% (*n* = 1,822). Our users also reported that the *Saathealth* intervention nudged them to change their behaviours. Using in-app surveys, we found that 80.9% of users reported buying more healthy food items for their children since installing the app (*n* = 47). Moreover, 70.2% of the surveyed respondents (*n* = 692) reported that they had increased their weekly consumption of protein-rich foods, such as lentils and eggs, for their children since installing the app. During the pilot phase of the app in 2019, we were able to nudge users to perform over 100,000 transactions for high-protein nutritious products worth over INR 2,100,000 (USD 30,000).

In the *Saathealth* family health intervention, our focus moved from creating awareness and improving knowledge levels to actually driving health and health services-seeking behaviour. This was achieved by running interactive social media campaigns, as outlined in Table 5, which not only imparted useful information on health and health savings behaviour but also nudged users to complete short surveys to understand their awareness levels and nature of their problems related healthcare access in different therapy areas, as well as connected them with service providers curated to their wallet sizes.

**Table 5:** A summary of targeted social media campaigns run between July 2022 to March 2023 to promote and assess health seeking behaviours.

Social media campaign type	Campaign duration (days)	Qualified leads/responses generated
An extensive survey to gather comprehensive information on the prevalence and awareness level about thyroid-related disorders	21	1,033
A detailed survey to assess the awareness levels about cancer among women	14	597
A lead-generation form designed to generate user interest in Diabetes care packages	30	6,426
An in-depth survey aimed at understanding awareness levels regarding Financial Literacy and the importance of planning for the financial burden associated with health emergencies	24	2,209
Promoting an affordable telehealth service specifically tailored for underserved health consumers	105	8,218
An awareness campaign to inform users about a health insurance product.	19	216

### 3.4. User satisfaction

We gathered qualitative feedback about the family health intervention from users; they reported that the app was easy to use and had useful information and advice that they could follow in their daily lives. Community health workers also reported that the app content was easy to understand, helping them better educate the community members. Table 6 displays the testimonials gathered from users of the Saathealth family health intervention, while Table 7 presents some of the reviews provided by users of the Saathealth family health mobile app on the Google PlayStore.

**Table 6:** Testimonials on the *Saathealth* family health mobile apps collected from user interviews.

App	Testimonial	User details
*Saathealth* family health app	“The app is very easy to use and videos have been made very simple, which is very easy to understand. There is a lot of important information given in this app which would be definitely helpful for parents.”	Community health worker
	“Thank you for giving us accurate information.”	Father in the age group of 20–30 years
	“It's really helpful for us to use video in our session and for parents’ groups. In a simple way they explained some topics very well…so it's easy for parents & kids to understand that particular topic.”	Community health worker

**Table 7:** Reviews provided by users of the Saathealth family health mobile app on the Play Store.

App	Reviews
Saathealth family health app	“Awesome app nice concept for kids and parents”
	“Smooth interactions with app. Highly self-explanatory”
	“Easy to use app…simple and informative videos.”
	“Very useful app with relevant health content.”
	“Animation & Hindi made it simple for all”

Users could earn points when they shared the mobile apps with their friends and family, and this gamification feature helped further expand the app's reach.

## 4. Discussion

In this case study, we demonstrate real-world evidence of successful scaling strategies for an mHealth intervention in a resource-limited setting. Till date, the *Saathealth* family health digital interventions, aimed at bridging the health information gaps prevalent in low-income populations, have cumulatively reached >10 million users across India. Through our experiences, we learnt that the following strategies were instrumental in helping our family health intervention achieve scale and sustainability:

• Ensuring that the interventions were designed in accordance with the researched user journey, and were culturally relevant.
• Using omni-channel approaches to deliver infotaining, gamified content.
• Delivering iterative product features.
• Testing and tailoring the interventions using real-time analytics, and
• Building partnerships to offer health services in the interventions.

These strategies not only helped us reach a large number of users but also boosted engagement levels and supported the long-term financial sustainability of the intervention, which in turn drove positive health behaviour changes. The subsequent sections discuss each strategy in detail and how these learnings can be applied in real-world settings by others.

### 4.1. Creating credible, infotaining content through ensuring localization and cultural relevance

One objective of the *Saathealth* intervention has been to address the scarcity of local language digital health content in India. Approximately 44% of Indians are Hindi-speakers; hence, our interventions aimed to meet the health information needs of a large proportion of Hindi-speaking, underserved Indians (17). Even when digital health content was available, it was often translated from original content written for an English-speaking audience, leading to missing cultural nuances and lack of consideration of local sensibilities (18). Our formative on-the-ground research among young families in low-income communities yielded invaluable insights that drove our content and product strategy (18). For example, we found that even though our target users were literate by definition, they were not comfortable with text-based digital content. Hence, we focused on creating relevant, infotaining, short-form animated videos that resonated with Indian traditional family values. Our close interaction with the target users also allowed us to gain unique insights into their family structures, lifestyles, and environments. Interviews and surveys allowed us to innovate and design interventions that meet the needs of the beneficiary families. These preliminary research exercises helped us design relatable characters and craft storylines that echoed traditional Indian familial values and covered practical problems associated with healthcare costs. This helped us design impactful user interfaces and create engaging storylines and characters for the videos. These exercises proved to be successful, as videos and reels were the most frequently consumed content types in the *Saathealth* intervention.

Our content production process ensures that the content is backed by credible scientific evidence since its inception. This process was also easily replicable, which was key in achieving scale as the content and product teams in the organisation evolved over time. Only the practical health tips need to be contextualised for cultural relevance, thereby allowing room for scale-up. Thus, localised and relevant content, created through formative research with the target users and robust scientific evidence review, is critical for making health messages impactful.

Given the constraints in conducting on-the-ground research in the wake of the COVID-19 pandemic, we ensured that the content in the family health intervention remained relevant through online interviews or phone calls with target users and partners. Through these interviews, we discovered that there are gaps in health insurance, thyroid disease, and mental health awareness. We administered surveys using Google forms and Facebook to gather more quantitative and qualitative data on knowledge gaps and practical needs related to these topics, and subsequently incorporate these learnings into our content plan.

### 4.2. Disseminating content using omni-channel approaches

Content trends and preferences are continually evolving, especially with the emergence of newer platforms and features such as TikTok, Instagram reels and stories, and YouTube shorts (19). Thus, mHealth interventions should keep up with these evolving trends to ensure they meet users' preferences. Our omni-channel approach involved different content delivery platforms (mobile app, YouTube, Instagram, and Facebook) and different content types (animated videos, doctor-delivered videos, quizzes, infographics, and notifications). During the development and deployment of the children's health app, a key transition that we observed among our target mobile users was the rising popularity of short-form videos. With apps such as TikTok and the expanding reach of YouTube, individuals were drawn to mobile-video content that was brief and easy to access (20). Parallel journeys on different social media channels allowed us to reach and build a community of interested users who could be guided and nudged to download the app.

Our focus on short-form, video-led content resulted in high engagement in the children's health intervention, with users having consumed >212 million seconds of content in 2019. The high content consumption on the children's health mobile app is attributable to the reporting period, which was a year after the launch of the intervention, allowing time for the intervention to scale and mature. User feedback about health content on the app showed that video formats optimised for small, low-quality displays were most effective in driving engagement, as they allowed for easier comprehension and retention of information.

We continued this video-led content strategy in the family health intervention by expanding to an omnichannel distribution approach, reaching families via multiple social media platforms in addition to the mobile app. We even converted our static infographics to dynamic infographics with voice overs. These short-form videos helped ramp up engagement, indicated by the high video views (5,145 views from February 2022 to June 2022). Instagram reels and YouTube shorts gained immense popularity in India after the ban of TikTok by the government in 2020 (21). We leveraged this popularity by publishing engaging short-form videos as reels, which further helped our content reach more potential users. Another advantage of Instagram reels and YouTube shorts is the ability to extract metrics such as plays, accounts reached, and likes, which all help monitor the scalability and reach of mHealth interventions. The family health intervention was also designed to launch on omni-channel platforms in order to significantly expand the reach to audiences across India. Engaging with users on social media also gave us a chance to pull back insights on the content we publish and allowed us to adapt the content themes based on real-time user feedback. Thus, a combination of social media platforms and a mobile app can help further scale and sustain mHealth interventions.

### 4.3. Gamification features for enhanced user experience

We integrated gamification to boost and sustain user engagement in the *Saathealth* interventions. Our gamification features included quizzes, leader boards, and games such as spin-the-wheel. The leaderboard serves as a social influence tool—it displays users' rankings based on engagement levels, knowledge levels (from the quiz responses), and content consumption. This feature is grounded in the “motivation” element of the Fogg Behavior Model, rewarding users for positive app behaviours and instilling in them a sense of achievement (15). Gamification is relatively nascent in the digital health field, but its potential in driving behaviour change is being increasingly recognized in mHealth interventions. In a recent systematic review exploring the correlation between gamification mobile apps and physical activity behaviours, gamification elements resulted in modest improvements in physical activity participation (22). The authors categorised the gamification elements as achievement and progression oriented (challenges, points, levels, leaderboards, rewards, goal-setting, feedback, progress bars, badges), social interaction oriented (competition, collaboration, social support), and immersion oriented (story or theme, avatars).

The *Saathealth* interventions primarily use achievement and progression oriented gamification features to sustain engagement. Users earn points for complete video watches and correct quiz responses, allowing them to climb up the leaderboards and redeem rewards. Gamification was built into the interface design through the quiz functionality. Quizzes are gaining attention as medical education tools that can help promote active engagement and healthy competition, while providing real-time feedback and insights on knowledge levels (23, 24). These quizzes were more than just a knowledge check; they allowed users to compete with others, earn points, progress from one level to the next, and enjoy the learning journey. This form of knowledge check also allowed users a safe space to test their learning and learn from their mistakes in a fun and safe manner. In our experience of running digital health interventions across varied audiences and geographies, quizzes were popular gamification features, as indicated by the 88,021 quiz attempts on the *Saathealth* family health mobile app in a 3-month time period. We attribute this increased engagement to the desire for instant gratification and notifications that helped drive an easy way to obtain points. We also linked app shares with points, motivating users to share the *Saathealth* mobile apps with their family and friends. Gamifying the app share process helped us further scale the *Saathealth* interventions and significantly lower our cost of user acquisition. Before we could build official partnerships in the early stages of the intervention, gamifying the app share process resulted in loyal users and helped scale the reach of the mobile app to geographies beyond what our online channels could target. This significantly lowered our cost of user acquisition.

Hence, based on our experiences, rewards-driven quizzes in mHealth interventions can be popular gamification tools that can help improve app engagement levels, and gamifying the share app feature can make users champions of mHealth interventions and lower overall acquisition costs.

### 4.4. Using real-time analytics to adapt the user's digital experience by using interactive content

Digital interventions provide the platform to reach health consumers at scale and enable them to “pull” the resources they need. More importantly, they provide the mechanism to monitor health consumer behaviour using real-time analytics. We set up a live dashboard to review anonymized user engagement and behaviour to learn from and iterate the intervention. These real-time insights allow us to test and iterate new content types and themes, product changes, and gamification elements on a monthly basis, helping us drive users towards products and services. Real-time analytics was an indispensable tool to test, tailor, and continuously iterate our solution. It enables us to produce high-volume content and nudges, which keeps users interested and creates “a-ha” moments for them.

Real-time analytics on family health digital intervention have allowed us to track the evolving needs of health consumers. We made changes to the registration process on the app to make it easier for users to experience the content offerings. We expanded our mental health content based on the high engagement with mental health and resilience content amongst our communities, as videos and quizzes in this category were among the most frequently consumed. Community feedback on specific themes such as thyroid health also provided us insights into specific patient concerns, allowing us to invite medical experts to address these in new content formats.

Our analytics also taught us how widely digital health interventions could travel if designed with the right sensibilities. Our family health intervention has been reaching users across 22 states and 91 cities in India, including large cities as well as tier-two towns

While the tools to track user engagement are available, we learnt that using these effectively requires considerable investment. Besides having dedicated resources to manage data analytics, our multidisciplinary team reviews the analytics collectively on a weekly basis to draw inferences and implications for product iteration, content updates, distribution, and partnerships. These learnings are drawn from the analytics in real time, making us responsible to respond in an agile manner as well. Health consumers have come to expect the same promptness and responsiveness they experience from other digital tools from their digital health interventions. This is an opportunity to harness real-time analytics to effectively respond to health consumer needs.

### 4.5. Sustainability models

Digital routes to reach and engage health consumers are a reflection of the increasing mobile adoption within target users in India. Our pilots demonstrate that these channels provide health product and service providers to reach consumers at a lower cost, offering the opportunity to redesign sustainable price offerings. Further, digital engagement also offers health providers the opportunity to better understand evolving behaviours in order to design tailored and targeted product offerings. This is vital in a country with diverse and evolving health behaviours. Finally, our experience shows that longitudinal digital engagement with users offers the means to extend lifetime consumer engagement, with a potentially positive impact on health outcomes as well the sustainability of the product and service offerings.

While exploring sustainability models, our team has focussed on health services and financial product providers as potential customers for our digital interventions' offerings due to the following challenges that they face due to limited insights on health behaviours and lack of platforms to provide longitudinal journeys to support health experiences. Due to this and the high cost of user acquisition, there are very few financial products designed to support the health needs of low-income users and the few that exist are largely being pushed out. There is a compelling need to educate and create a pull from the consumers. The health and financial literacy awareness social media campaigns summarised in Table 5 convinced us that there is a need for health services and financial products amongst the target online communities that can serve as a demand generation channels for supply-side partners.

### 4.6. Future implications

The limited availability of health workers in low- and middle-income countries (LMICs) necessitates scalable digital interventions to address the growing burden of non-communicable diseases (NCDs) and infectious diseases. Mobile devices' ubiquity in LMICs presents an opportunity to explore the role of mHealth solutions in alleviating this burden. Our work has shown that health consumers in low-income communities are receptive to engaging with digital interventions tailored to their health needs.

Delivering preventive and engaging health information through scalable digital platforms can reduce the health education workload on traditional health workers, allowing them to focus on other priorities. Directly reaching health consumers empowers them to access critical health information at their convenience, independent of health workers. In addition, the Saathealth family health intervention offers a digital and scalable platform to reach, educate and empower low-income families with reliable information to facilitate their financial decision-making regarding health, enabling not just positive health outcomes but also economic resilience to handle the burden of healthcare expenses.

Engagement on digital platforms also enables real-time feedback, facilitating prompt intervention iterations to address community needs. Our work offers insights into using scalable digital platforms for population-wide behaviour change interventions. The WHO MAPS toolkit, combined with our scaling strategies, can significantly enhance the reach and effectiveness of mHealth interventions in resource-limited settings.

## 5. Conclusions

By leveraging the high penetration of digital tools in an underserved population and deploying personalised content to targeted audiences through the *Saathealth* digital health interventions, we were able to reach underserved communities with critical health information to drive preventive behaviours. The future implications of this work can mean improved access to primary healthcare, timely health-seeking behaviours, and improved health outcomes. In a short span of 2 years since launch, the family health intervention will also be able to demonstrate how a sustainable business model can create scalable impact with underserved health consumers.

## Data Availability

The raw data supporting the conclusions of this article will be made available by the authors, without undue reservation.
